# Bacteriology Run Mad

**Published:** 1898-02

**Authors:** 


### BACTERIOLOGY RUN MAD.

The Berlin correspondent of the *Standard* telegraphs this great discovery by a German professor: "Professor Marpmann, of Leipsic, has recently examined sixty-seven kinds of ink such as are used in schools, and has arrived at results, which show that it may, under certain circumstances, be dangerous to wound oneself with an inky pen. Most of the inks he examined were made of gallnuts, and contained a large quantity of micrococci bacteria and fungi. One sample, made of nigrosine, an aniline pigment, and taken from a newly opened bottle, also contained quantities of fungi and bacilli. From a red and a blue sample the Professor cultivated a bacillus which killed a mouse in four days; but they were taken from bottles which had been open for three months."

Is there any known substance, we wonder (says the *Westminster Gazette*), in which a German professor could not cultivate a bacillus which would kill a mouse in four days? It seems to us rather creditable to the mouse to have lived four days. The moral is: Don't drink ink, or wipe your pen on your lips. The temptation to do these things is so besetting that these cautions ought to be widely disseminated. By dint of great determination we have ourselves resisted the impulse to take a draught of ink in the early morning.—*Food and Sanitation.*

